# Patients’ Perspective on Their Experience of Dental Treatments Covered by Public Health Insurance in Romania—A Pilot Study

**DOI:** 10.3390/ijerph19010272

**Published:** 2021-12-27

**Authors:** Mariana Cărămidă, Ana Maria Cristina Țâncu, Marina Imre, Mihaela Adina Dumitrache, Christina Mihai, Ruxandra Sfeatcu

**Affiliations:** 1 Department of Oral Health and Community Dentistry, Faculty of Dental Medicine, “Carol Davila” University of Medicine and Pharmacy, 17–21 Calea Plevnei Street, Sector 1, 010221 Bucharest, Romania; mariana.caramida@umfcd.ro (M.C.); mihaela.dumitrache@umfcd.ro (M.A.D.); ruxandra.sfeatcu@umfcd.ro (R.S.); 2 Department of Complete Denture, Faculty of Dental Medicine, “Carol Davila” University of Medicine and Pharmacy, 17–21 Calea Plevnei Street, Sector 1, 010221 Bucharest, Romania; marina.imre@umfcd.ro; 3 Department of Preventive Dentistry, Faculty of Dental Medicine, “Carol Davila” University of Medicine and Pharmacy, 17–21 Calea Plevnei Street, Sector 1, 010221 Bucharest, Romania

**Keywords:** oral health, dental insurance, patients’ perspective, oral health coverage

## Abstract

Although the aims of any public health coverage are prevention, diagnosis, treatment, rehabilitation, and maintenance, dental services are hardly ever included in services. The goal of our pilot study is to assess the perspective of a group of adult patients on their covered dental treatments. The present cross-sectional study was conducted on 140 patients who reported their perception by filling in a questionnaire. All the collected data were statistically analyzed using IBM SPSS Statistics 25. Most of the subjects (40.7%, *n* = 57) were treatment oriented, visiting the dentist only in an emergency situation. A total of 40.7% (*n* = 57) of the participants stated that all the dental treatments had coverage and 22.8% (*n* = 13) had to split their treatment plan because of the insurance budget limit. The subjects who had chosen covered dental services because they considered it was a right they should benefit from (53.7%, *n* = 22) and those who had chosen covered dental services because of financial reasons (29.3%, *n* = 12) were more frequently unsatisfied with the types of covered dental services. The reduced level of satisfaction was associated mainly with the list of dental procedures accepted for coverage and also with younger and highly educated patients. For a more accurate description, the present study should be completed by future studies not only on a representative population at national level, but also by assessing the perspective of dental professionals.

## 1. Introduction

Oral health diseases are among the most frequently met health issues at a global level, even though dental caries and periodontal disease are highly preventable [1]. Untreated dental caries on permanent dentition remains the most common health condition for the global population [2] and its complications lead to disabilities that significantly affect the quality of life [3]. Moreover, periodontitis, a complex and chronic inflammatory disease and a threat to general health, is the sixth most prevalent health issue at a global level and the main reason for tooth loss [4]. Unfortunately, oral diseases are as expensive to treat as they are frequent. A recent report states that in 2020 the spending for the treatment of oral diseases represented 30% of the total health expenditure within European countries from OECD (Organization for Economic Cooperation and Development) [3], while, according to previous economic reports from 28 European Union countries, in 2015, dental diseases were the third most expensive health condition to treat, surpassed only by diabetes mellitus and cardio-vascular diseases [5].

There is a well-known connection between one’s socio-economic status and oral diseases, meaning that individuals with lower incomes are more frequently affected by oral diseases [6]. Likewise, their access to dental services is limited since the out-of-the-pocket expenditure represents a great financial effort to cover the costs for dental treatments, thus increasing the gap between the treatment needed and the treatment offered [7,8]. A higher level of socioeconomic inequalities regarding attendance and use of dental services was observed among patients from countries where the public health care system does not cover dental procedures, compared to patients from countries with these benefits available to a certain degree [9].

Although dental caries and periodontal disease are highly preventable with appropriate patients’ behavior (such as oral hygiene, balanced diet with a reduced consumption of carbohydrates as well as smoking avoidance or cessation), preventive strategies also include the support from oral health care services for maintenance, early diagnosis, and treatment [10]. The objectives of the public health insurance-based coverage of the healthcare services are prevention, diagnosis, treatment, rehabilitation, and maintenance. Yet, dental services are hardly ever included in the benefits of public health insurance. However, including dental care services is both righteous and efficient for important reasons: epidemiological, economic, rightly, correlations with the systemic health, and coalition with other professionals in medical fields [7].

Similar to many European countries, in Romania the public health system offers insurances that are funded through the national taxation system. Health insurance is guaranteed for children and youngsters under 18 years old, employees, and those who are retired, and it covers the health services in all public medical institutions as well as in those private institutions that opt for a collaboration with the National Health Insurance House for reimbursement. When it comes to the oral healthcare field, in Romania, the vast majority of dental clinics are private and the specific collaboration for dental treatment reimbursement is not mandatory. A report published in 2015 about the Romanian healthcare system and provision of dental services stated that only 2613 Romanian dental offices, out of a total number of almost 13,000 of all dental offices registered at national level, were contracted in 2015 with the NHIH for oral healthcare reimbursement in 2015 [11]. Dental institutions that work under contract with the National Health Insurance House (NHIH) are offered a fixed set of dental procedures as follows: preventive treatments (annual examination and professional cleaning (once a year for adults and twice a year for children under 18 y.o.)), fissure sealing (one every 2 years), topical fluoridation (for children between 6 y.o. and 14 y.o.), conservative treatments (fillings), endodontic treatments, periodontal treatment (non-surgical), fixed prosthetic treatments (resin/metal-resin crowns), removable prosthetic treatments (acrylic dentures (one every 4 years)), orthodontic treatments (removable, space maintainers, functional appliances), surgical treatments (extractions, splint after traumatic dental lesions, TMJ repositioning), and oral pathology treatments (specific oral mucosa lesions). Moreover, there are fixed prices established by the NHIH for these dental procedures and the percent of coverage is either 100% for children under 18 years old, students under 26 years old and adults who benefit from special social security rights, or 60% for employed or retired adults. The standard budget offered by the NHIH for dental care services is limited to 2000 Romanian Lei (RON) (approx. 400 EUR, about the value of the gross minimum wage in Romania) per month per general dentist, a budget that is increased by 50% for a general dentist that works in rural areas, on the one hand, and by 20% for a dentist specialized in a specific field of dentistry. The terms and conditions in the contract offer the possibility for children and adults to benefit from basic oral health services, with no or reduced costs, aiming the maintenance or regain the functionality. However, the fixed list of eligible treatments does not include up-to-date dental treatments commonly and widely used nowadays, such as fiber-reinforced posts, metal–ceramic or all-ceramic restorations, surgical treatment of periodontitis or microscopically assisted endodontic treatments, or dental implants. On the other hand, this limited monthly budget leaves the chance for coverage to a reduced number of procedures and patients, therefore many cases of unmet dental needs with patients that cannot afford dental treatments paid out-of-the-pocket.

The aim of the study is to assess the experience of covered dental treatments of a group of Romanian patients as a starting point for establishing the need for favorable public health policies conductive to dental treatments using the health insurance system.

## 2. Materials and Methods

### 2.1. Survey Methodology

The present cross-sectional study was conducted in October–November 2021 by a group of academic staff from the Faculty of Dental Medicine of the “Carol Davila” Medicine and Pharmacy University, Bucharest, Romania. The survey was designed to be a pilot study on a group of adult patients in Bucharest, the capital city of Romania, and to be continued further by extending it to different regions, residential areas and age groups at a national level. In the present survey, the evaluation of patients’ attitude and behavior regarding previous experiences of coverage of their dental treatments was performed by using a self-administered online questionnaire that was distributed and available for completion on electronic devices in two dental clinics in Bucharest, Romania. The study was implemented under the protocol approved by the Ethics Commission of the Scientific Research of the “Carol Davila” University of Medicine and Pharmacy in Bucharest, Romania, with ethical approval no. 28446/18.10.2021. Subjects selected to participate in the study were invited to fill in the questionnaire and were informed about the survey in respect to the Declaration of Helsinki and the current European privacy law, highlighting, in an introduction section of the questionnaire, the scientific aim of the study, that the questionnaire was anonymous and as to their right to interrupt the completion of the form at any moment in case of withdrawal. All subjects invited to participate to the study agreed to take part in it and expressed their consent by completing the survey. No personal data were collected through the form and, as an anonymous web-survey, no sensitive data were collected.

### 2.2. Survey Population

The sample comprised subjects selected from the patient pool of two dental clinics in Bucharest, using simple random sampling as the selection method: from all patients who had appointments between 19 October and 2 November, and were eligible to participate, according to the established inclusion and exclusion criteria, we used the random number method to select the subjects. All selected patients agreed to participate. The inclusion criteria were adults (>18 years old), with at least one dental procedure in the past year that had been covered by the National Health Insurance, treated in Bucharest, irrespective of the type of dental care unit (public/private, dental office/hospital/university). The exclusion criteria were dental services workers (dentists/dental assistants) or dental students.

The sample was made up of 140 adults, of average age of 52.73 (SD 20.67 years, range 18–92, median of 50 years). Most of the analyzed participants were women (61.4%, *n* = 86) employees (58.6%, *n* = 82) or retired (31.4%, *n* = 44), and with an educational background of either secondary (50.7% (*n* = 71)) or tertiary education level (49.3% (*n* = 69)) (Table 1).

### 2.3. Survey Questionnaire

The questionnaire used for the assessment was formed of 12 items represented by both open-ended and single/multiple-choice questions, referring to 3 main aspects: (1) oral hygiene and dental attendance habits, (2) opinions and behaviors related to their history of dental treatments covered by the public health insurance and (3) socio-demographic data (Table S1).

In the section referring to their previous dental treatments covered by the national health insurance, the questions included aimed to evaluate: the type of previous dental treatments covered, the possible limitations regarding the dental treatment plan or time frame due to conditions required by the public health insurance system and the level of satisfaction regarding the coverage experience.

The estimated fill-in time for the questionnaire was 5 min.

### 2.4. Data Analysis

All the data in the study were analyzed by using IBM SPSS Statistics 25 (IMB Corp, Armonk, NY, USA). Quantitative variables were tested for normal distribution using the Shapiro–Wilk Test and were written as averages with standard deviations or medians with interquartile ranges. Qualitative variables were written as counts or percentages. Quantitative independent variables were tested using Mann–Whitney U/Kruskal–Wallis H Tests, according to their non-parametric distribution. Qualitative variables were tested using Fisher’s Exact tests/Pearson Chi-square tests.

## 3. Results

In the studied group, most of the subjects were treatment oriented, 57 (40.7%) visiting the dental office only in need (when symptoms arise) and 34 (24.3%) rarely, once every few years; moreover, the most frequently mentioned reasons for the latest dental attendance were self-assessed dental treatment needs (55%, *n* = 77) or emergency/dental pain (31.4%, *n* = 44) (Table 2).

Regarding the previous dental treatments that were covered by the national health system, the most frequently mentioned by the subjects were direct restorations (45.7%, *n* = 64), dentures (25%, *n* = 35), dental extractions (22.1%, *n* = 31) and root canal treatments (20, *n* = 28). When it came to the budget available and the total costs of their needed dental treatments, only 40.7% (*n* = 57) of the participants said that all the dental treatments were covered and most of them said that all the treatments were performed as scheduled with no delays to fit in the budget (73.7%, *n* = 42). Only 22.8% (*n* = 13) had to split their treatment plan across multiple months because of the insurance budget limit. Of those who answered negatively, most of them said that they needed complex procedures that could not be covered by the insurance budget (67.5%, *n* = 56) (Table 3).

Most of the participants declared they chose dental treatments with included coverage because they considered it was the standard procedure (44.3%, *n* = 62) or because it was their right (32.9%, *n* = 46). In terms of the level of satisfaction regarding the experience that involved the use of public health insurance for previous dental treatments, most of the participants were pleased with the covered dental services, especially with the quality of the treatments (88.6%, *n* = 124), the necessary formalities for coverage (82.9%, *n* = 116), the medical office environment (82.1%, *n* = 115) and the waiting time for coverage (77.1%, *n* = 108) (Table 4).

**Figure 1:**
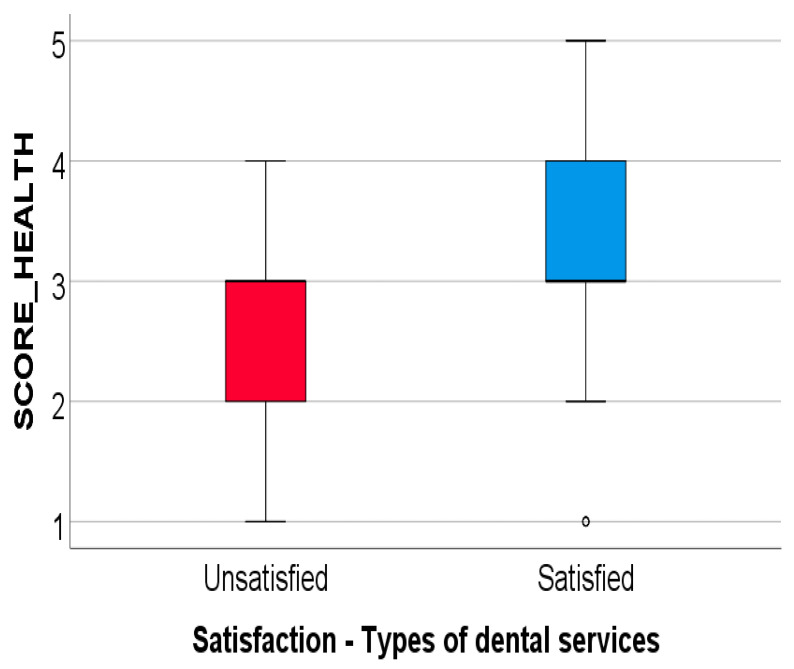
Comparison of oral health score between answers according to satisfaction about the types of covered dental services. In the boxplot figure, the dot (°) represented above the “Satisfied” text, represents a value which SPSS considers a potential outlier. In this case the value represents a low potential outlier (a value which was more than1.5 IQR but at most 3 IQR below quartile 1 of the represented score).

Subjects were asked to self-assess their oral health status and the following answers were observed: excellent 1.4% (*n* = 2), very good 25% (*n* = 35), good 49.3% (69), satisfactory 20% (*n* = 28) and very poor 4.3% (*n* = 6). A score for measuring the oral health (described by the participants) was constructed (SCORE_HEALTH), with a range from 1 point (very poor) to 5 points (excellent). The average score is 2.99 ± 0.827 points with a median of 3 points (indicating a moderate level of oral health). The data from Figure 1 show the comparison of the oral health score (SCORE_HEALTH) between the two subgroups of participants based on their declared satisfaction regarding the type of dental treatments eligible for coverage from the public insurance. The score has a non-parametric distribution according to the Shapiro–Wilk test (*p* < 0.05). According to the results, the difference observed between the satisfied and the dissatisfied patients is statistically significant (*p* < 0.05) and according to Dunn–Bonferroni post-hoc tests, participants who declared satisfied about the types of covered dental services had a significantly higher oral health score (median = 3 points, IQR = 3–4 points) compared to the participants who were dissatisfied (median = 3 points, IQR = 2–3 points) (*p* = 0.001).

**Figure 2:**
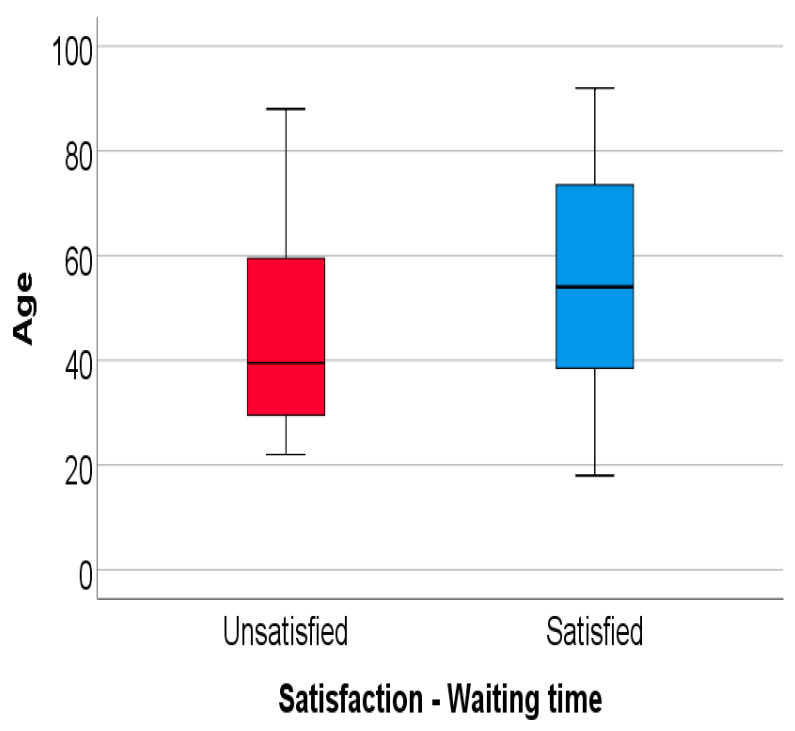
Comparison of age between answers according to satisfaction about the waiting time for coverage.

Moreover, the data from Figure 2 show the comparison of age between the patients who were or were not satisfied about the waiting time for coverage. The age has a non-parametric distribution in both groups according to the Shapiro–Wilk test (*p* < 0.05). According to the results, the participants who declared themselves satisfied about the waiting time had a significantly higher age (median = 54 years, IQR = 38.25–73.75 years) in comparison to the participants who were not satisfied with it (median = 39.5 years, IQR = 29.25–59.75 years) (*p* = 0.010).

When the data regarding the distribution of the participants according to gender and certain variables in the survey were analyzed, we noticed statistically significant differences between groups. Participants who wanted covered dental services because of financial reasons (out of 136 valid answers) were more frequently women than men (23.8% vs. 7.7%) (*p* = 0.020). In addition, participants who reported that all services were covered by the health insurance authority were also more frequently women than men (48.8% vs. 27.8%) (*p* = 0.014) (Table 5).

Regarding the connection between the educational level and the frequency of dental visits as well as the satisfaction with the waiting time for the dental treatments to be offered with coverage from the public health insurance, the results show statistically significant differences as follows (Table 6):

- Participants who visit the dentist when needed (when problems arise) were more frequently associated with a secondary education level (52.1% vs. 28.2%), while participants who visit the dentist more than one time each year were more frequently associated with a tertiary education level (40.6% vs. 19.7%) (*p* = 0.008);
- Participants who considered themselves satisfied about the waiting time for coverage were more associated with a secondary education level (84.5% vs. 69.6%) (*p* = 0.044);

Furthermore, regarding dental attendance characteristics (frequency and reasons for dental visits) in correlation with aspects related to the use of insurance for dental services, according to the results, all the differences observed between groups are statistically significant and show as follows:

- Participants who considered themselves satisfied with the dental treatment quality (when health insurance was used) were more frequently associated with visits to the dentist when needed (when problems arose) (96.5% vs. 78%), while participants who did not consider themselves satisfied were more frequently associated with one visit or less at the dentist (22% vs. 3.5%) (*p* = 0.016) (Table 7).
- Participants who declared that all dental services were covered by the health insurance authority were more frequently associated with routine dental check-ups (76.9% vs. 37.7%), while participants who did not benefit from the coverage for all their necessary dental treatments were more frequently associated with pain/emergency as reasons for dentist visits (62.3% vs. 23.1%) (*p* = 0.033) (Table 8).

The data in Table 9 show the distribution of the participants according to their satisfaction about the types of previous covered dental services and certain aspects regarding their motivation behind the use of public health insurance for dental treatments as well as whether all dental services benefitted from coverage. Results show statistically significant differences as follows:

- Participants who had chosen covered dental services because of their dentist suggestion were more frequently unsatisfied about the types of covered dental services (43.9% vs. 7.4%) (*p* < 0.001);
- Participants who had chosen covered dental services because they considered that it is a right they should benefit from were more frequently dissatisfied about the types of covered dental services (53.7% vs. 25.3%) (*p* = 0.003);
- Participants who had chosen covered dental services because of financial reasons were more frequently dissatisfied with the types of covered dental services (29.3% vs. 12.6%) (*p* = 0.027);
- Participants who considered that all services were covered by the health insurance authority were more frequently satisfied about the types of covered dental services (50% vs. 20.5%) (*p* = 0.001).

In terms of distribution of the participants according to their satisfaction with the waiting time for coverage and their motivation of the use of public health for coverage, statistically significant differences were observed and support that participants were more frequently dissatisfied with the waiting time when they had chosen covered dental services because of their dentist suggestion (51.7% unsatisfied vs. 9.3% satisfied, *p* < 0.001) or because of financial reasons (31% unsatisfied vs. 14% satisfied, *p* = 0.033) (Table 10).

## 4. Discussion

According to World Health Organization reports [6], at a global level, the percentage of coverage for oral health services offered by different countries varies between 40% and 80% for adults and there is an inverse correlation between the national income level and the mean oral health care spending through public health insurance. The current percentage of 60% in Romania is characteristic for low-to-middle income countries [6]. The World Health Organization also advocates for universal dental coverage, as part of universal health coverage, in order to facilitate the access to oral health care services for large populations, irrespective of their incomes. In other words, appropriate dental insurance should be designed to remove the cost barriers that could impede people to access dental care services [6]. A report on oral health status and policies in European countries [12] points out the differences in frequency of dental visits between populations from regions in Europe with different economic background and different level of public funding of oral healthcare services, and offers as example the comparison between two opposite situations: one is from Catalonia, a country with high economic status, but almost inexistent dental services provided through public funds (only 5% of dentists work in public dental institutions where adults have free access only to oral surgical treatments [13]), where only one third of population visited the dentist in 2006; the second one is from Belarus, where the population’s access to dental services is still predominantly through public health system and where two thirds of the patients went to the dentist at least once in 2007. Moreover, outside Europe, in comparison to a study conducted in Canada, where among the subjects enrolled who had dental insurance, 82% visited a dental office in the past 12 months, in the present study on the Romanian population, only 35.2% of the patients declared attending a dental office at least once a year. Moreover, only 10.3% of the insured Canadian patients declared requesting dental treatment only for emergency, while the percentage met among the Romanian patients was three times higher (32%) [8].

When it comes to the most frequently needed dental treatments, we noticed differences between studied Romanian population and other European countries. In Western and Scandinavian countries [12], patients older than 50 years tend to visit the dental office more frequently and to request more preventive procedures (routine check-ups and maintenance treatments) than operative treatments. Romanian participants involved in the present study, from the aforementioned age category, are more curative oriented, as seen in the top five most frequently covered dental treatments that include invasive restorative and surgical treatment (endodontic treatments, removable prostheses, extractions).

In some countries in Europe, such as Germany and France, public health insurance offers favorable conditions predominantly for coverage of direct restorations [14], which was, as well, among the studied Romanian group, the most frequently met dental treatment covered by the public health insurance. Regarding the list of dental services eligible for coverage in Romania, more than half of the participants declared that not all their dental treatments were covered, mainly because the needed procedures were not on the eligibility list. This is a reason for which the rate of declared satisfaction was lower compared to other assessed aspects in our study. In comparison to the conditions of coverage in Romania, in Germany, in addition to the full coverage for a set of basic procedures, complex dental treatments, such as dental, surgical periodontal treatments or ceramic crowns, are included among the eligible treatments for coverage, with a fixed amount supplied by the insurance and the rest to complete the dental costs paid by the patient [15]. Moreover, in Romania the monthly budget offered per dentist is limited to EUR 400, and this is the reason why one quarter of the participants of the present study declared they needed to split their dental treatment on multiple months to fit in the budget. Another quarter claimed that they had to pay out-of-the pocket because the total costs of their dental treatments were over the available monthly budget. In Germany, because there is no upper budget limit offered per patient and 99% of the dentists work under contract with the national health insurance system (SHI), 80% of the adults use the oral healthcare system annually [15]. In France [16], there is no limited annual budget per dentist or patient, but the public insurance system covers 70% of the fixed imposed costs for basic preventive and operative dental treatments and offers a fee for a larger list of prosthetic and orthodontic treatments leaving the dentists to impose their costs to the patients. Dental implants and periodontal surgeries are excluded by the public insurance. However, patients have the opportunity to use the complimentary health insurance that is not mandatory and contracted by either individual or employers and used to compensate the percentage of medical costs not covered by the public system. Thus, in 2013 in France, the total spending for oral healthcare services were covered by the patients out-of-the pocket as little as 25.3%, with a more consistent contribution through either public health insurance system (32.1%) or complimentary health insurance system (39.3%) [16]. Greece, on the other hand, reports a percent of 96% of expenditure for dental services supported by the patients [17], similar to Italy, where the public funding for oral health related spending represents only 5–8% of the total costs [18].

In Poland, the country that has the most similar public health insurance system for oral healthcare field to Romania (in terms of the eligible dental treatment as well as contracting conditions for dentists working in the private sector), only 15% of the patients use oral healthcare through public funding [19]. Among the Romanian participants of the present study, the main reason for choosing covered dental treatments (in addition to the fact that it was the standard method of payment in the dental units where they were treated) was their awareness of this right, according to Romanian patient law, that they have and should benefit of. Therefore, policy makers should view the opportunity of coverage for the population not only of the aspect related to costs, but also of the personal values important for the population.

Patient-reported outcomes, which express patients’ perception on their health status, experience with healthcare system and satisfaction with the provided health-related procedures, are encouraged and taken into consideration in the U.S.A. This aims to improve the reimbursement payment system through changing the traditional fee-for-service method with pay-for-performance reimbursement and this contributes to a concept known as value-based purchasing [20].

According to the most recently published report [21] on Romanian health-related profile (in the context of the European health policies, systems and status), when it comes to oral health, in 2017 4.3% of the investigated Romanian patients declared unmet need for dental examinations because of three major reasons: costs, waiting time and traveled distance needed to access dental services. This reported rate is higher than the European Union medium rate (only 2.7%), but lower than the percentage of unsatisfied participants in the present survey when it comes waiting time (22.9%). However, our pilot study was limited to Bucharest, the capital city of Romania, where, although it does not concentrate the highest number of dental offices working in contract with the National Health Insurance House, it hosts the higher number of dentists [11]. Therefore, for an accurate and current overview at the national level, the present research should be continued by further assessment adapted accordingly, on larger and more diverse samples.

## 5. Conclusions

In the studied group, despite the fact that the participants had access to dental services either complimentary or at reduced costs through their public health insurance, one third of them chose to visit the dental office only in cases of dental pain/emergency.

Direct restorations were the most frequently covered procedures. Coverage of all needed dental treatments was rarely observed, some of the patients having to extend their treatment across multiple months due to the distribution of the budget from the public health insurance, while others had to cover the dental costs out-of-pocket, particularly because of the fact that part of the treatments was not eligible.

The reduced level of satisfaction was associated mainly with the list of dental procedures accepted for coverage, especially among younger and higher educated patients. When used for financial reasons, lack of satisfaction was observed among women and related to eligible dental treatments and waiting time.

Patients’ perceived experience and satisfaction with the oral healthcare reimbursement system, as final beneficiaries of the provided dental services, are of the outmost importance for taking future improvement measures able to respond to their current dental needs. However, for a more accurate description, the present research should be completed by another future research not only by extending it on representative population at the national level, but also by assessing the perspective of dental professionals, who are at the forefront of the oral healthcare system, as providers of dental care to patients, irrespective of the payment and reimbursement methods.

## Figures and Tables

**Table 1:** Demographic characteristics of the analyzed participants.

Parameter (*N* = 140)	Value
Age (Average ± SD, Median (IQR), Min-Max)	52.73 ± 20.67 years, 50 (34.25–70.75), 18–92
Gender (Nr., %)	86 (61.4%) Females 54 (38.6%) Males
Education level (Nr., %)	71 (50.7%) Secondary 69 (49.3%) Tertiary
Employment status on which public health insurance is offered (Nr., %)	14 (10%) Students 82 (58.6%) Employees 44 (31.4%) Retired

**Abbreviations:***N* = number (of participants with valid values); SD = standard deviation; IQR = interquartile range (written as a range: quartile 1–quartile 3); Min = minimum value; Max = maximum value; Nr. = number (of participants); % = valid percentage (of participants).

**Table 2:** Patients’ habits related to dental attendance.

Question	*N* (%)
**Frequency of Dental Visits**	
When needed, when problems arise	57 (40.7%)
Once every few years	34 (24.3%)
Once a year	7 (5%)
Twice a year	17 (12.1%)
Several times a year	25 (17.9%)
**The Main Reason for The Latest Dental Visit**	
Treatment/treatment follow-up	77 (55%)
Pain/urgency	44 (31.4%)
Routine check-up	13 (9.3%)
No answer	6 (4.3%)

**Table 3:** Patients’ history of dental treatments using public health insurance.

Question	*N* (%)
**Previous Dental Treatments Covered by the Public Health Insurance**	
Fillings	64 (45.7%)
Dentures	35 (25%)
Dental extraction	31 (22.1%)
Root canal treatment	28 (20%)
Dental crown/bridge	24 (17.1%)
No answer	11 (7.9%)
Periodic check-up	8 (5.7%)
Periodontal treatment	6 (4.3%)
Braces	3 (2.1%)
Oral Maxillofacial surgery	2 (1.4%)
**All The Dental Treatments Covered by The Health Insurance Authority**	
No	83 (59.3%)
Yes	57 (40.7%)
**If YES, Reasons Mentioned:**	
I covered all the treatments in the insurance budget and it was not necessary to wait for a period for the treatment to start	42 (73.7%)
I set the treatments across multiple months so that I could be limited to the insurance budget	13 (22.8%)
I was not aware that there was an insurance budget	2 (3.5%)
**If NO, Reasons Mentioned:**	
I needed complex procedures and some of them were not covered	56 (67.5%)
The treatments I needed were over the insurance budget	21 (25.3%)
I was not informed about the list of covered dental services	6 (7.2%)

**Table 4:** Patients’ attitude regarding previous dental treatments using public health insurance.

Question	*N* (%)
**Reasons for Choosing Dental Treatments with Included Coverage**	
It was the standard procedure	62 (44.3%)
It was my right that I wanted to benefit from	46 (32.9%)
It was the dentist’s suggestion	25 (18.4%)
Financial reasons	24 (17.1%)
No answer	4 (2.9%)
I have more confidence in these services	3 (2.1%)
**Satisfaction Regarding Various Aspects of The Covered Dental Services**	
The quality of the treatments	124 (88.6%) Yes 16 (11.4%) No
The medical office environment	115 (82.1%) Yes 25 (17.9%) No
The necessary formalities for coverage	116 (82.9%) Yes 24 (17.1%) No
The waiting time for coverage	108 (77.1%) Yes 32 (22.9%) No
The types of covered dental services	96 (68.6%) Yes 44 (31.4%) No

**Table 5:** Distribution of the participants according to gender and certain answers in the survey.

Answers (*N*, %)		Gender		*p* ^1^
		Female	Male	
Reason for covered dental services—Financial	No	64 (76.2%)	48 (92.3%)	0.020
	Yes	20 (23.8%)	4 (7.7%)	
Were all the dental treatments covered by the health insurance authority?	No	44 (51.2%)	39 (72.2%)	0.014
	Yes	42 (48.8%)	15 (27.8%)	

^1^ Fisher’s Exact Test.

**Table 6:** Distribution of the participants according to education level and certain answers in the survey.

Answers (*N*, %)		Education Level		*p* ^1^
		Secondary	Secondary	
Frequency of dental visit	When needed	37 (52.1%)	20 (29%)	0.008
	≤1 visit/year	20 (28.2%)	21 (30.4%)	
	>1 visit/year	14 (19.7%)	28 (40.6%)	
Satisfaction—Waiting time	Unsatisfied	11 (15.5%)	21 (30.4%)	0.044
	Satisfied	60 (84.5%)	48 (69.6%)	

^1^ Fisher’s Exact Test.

**Table 7:** Distribution of the participants according to frequency of dentist visit and certain answers in the survey.

Answers (*N*, %)		Frequency of Dental Visits			*p* ^1^
		When Needed	≤1 Visit/Year	>1 Visit/Year	
Satisfaction—Treatment quality	Unsatisfied	2 (3.5%)	9 (22%)	5 (11.9%)	0.016
	Satisfied	55 (96.5%)	32 (78%)	37 (88.1%)	

^1^ Fisher’s Exact Test.

**Table 8:** Distribution of the participants according to reason for dentist visit and certain answers in the survey.

Answers (*N*, %)		Reason for Dental Visits			*p* ^1^
		Routine Check	Pain/Emergency	Treatment/Treatment Follow-Up	
Were all the dental treatments covered by the health insurance authority?	No	3 (23.1%)	48 (62.3%)	26 (59.1%)	0.033
	Yes	10 (76.9%)	29 (37.7%)	18 (40.9%)	

^1^ Fisher’s Exact Test.

**Table 9:** Distribution of the participants according to the satisfaction about the types of covered dental services and certain answers in the survey.

Answers (*N*, %)		Satisfaction—Types of Services		*p* ^1^
		Unsatisfied	Satisfied	
Reason for covered dental services —Dentist’s suggestion	No	23 (56.1%)	88 (92.6%)	<0.001
	Yes	18 (43.9%)	7 (7.4%)	
Reason for covered dental services —Right to benefit of it	No	19 (46.3%)	71 (74.7%)	0.003
	Yes	22 (53.7%)	24 (25.3%)	
Reason for covered dental services	No	29 (70.7%)	83 (87.4%)	0.027
—Financial	Yes	12 (29.3%)	12 (12.6%)	
Were all the dental treatments covered by the health insurance authority?	No	35 (79.5%)	48 (50%)	0.001
	Yes	9 (20.5%)	48 (50%)	

^1^ Fisher’s Exact Test.

**Table 10:** Distribution of the participants according to the satisfaction about the waiting time for coverage and certain answers in the survey.

Answers (*N*, %)		Satisfaction—Waiting Time		*p* ^1^
		Unsatisfied	Satisfied	
Reason for covered dental services —Dentist’s suggestion	No	14 (48.3%)	97 (90.7%)	<0.001
	Yes	15 (51.7%)	10 (9.3%)	
Reason for covered dental services —Financial	No	20 (69%)	92 (86%)	0.033 ^1^
	Yes	9 (31%)	15 (14%)	

^1^ Pearson Chi-Square Test.

## Data Availability

The data presented in this study are available from the corresponding authors upon reasonable request.

## Supplementary Materials

The following are available online at https://www.mdpi.com/article/10.3390/ijerph19010272/s1, Table S1: Questionnaire on patients’ perception on their experience of dental services utilization with the use of public health insurance.
