# Supplementary material for: Patients’ Perspective on Their Experience of Dental Treatments Covered by Public Health Insurance in Romania—A Pilot Study
Source: Int J Environ Res Public Health. 2021 Dec 27;19(1):272. doi: 10.3390/ijerph19010272 (PMC8744563; doi:10.3390/ijerph19010272)
Supplement: Supplementary file 1 [file ijerph-19-00272-s001.zip › ijerph-1502773-supplementary.pdf]

Table S1. Questionnaire on patients´ perception on their experience of dental services utilization with the use of public health insurance

Question	Parameter	Questions, Response Scale and Romanian Translations (italic)	
**Q1**	Frequency of dental visits	How often do you visit the dental office?	*Cât de frecvent mergeți la dentist?*
		1. Several times a year<br>2. Twice a year<br>3. Once a year<br>4. When needed, when problems arise<br>5. Once every few years<br>6. Never	*1. De câteva ori pe an*<br>*2. De 2 ori pe an*<br>*3. O dată pe an*<br>*4. La nevoie, când apar probleme*<br>*5. O dată la câtiva ani*<br>*6. Niciodată*
**Q2**	Reasons for dental visits	What was the reason for your last dental visit?	*Care a fost motivul ultimei vizite la dentist?*
		1. Pain/emergency<br>2. Treatment/treatment follow-up<br>3. Routine check-up<br>4. I don't know	*1. Durere/urgență*<br>*2. Tratament/urmărirea tratamentului*<br>*3. Control de rutină*<br>*4. Nu știu*
**Q3**	Status that offers public health insurance	What is your current employment status on which you benefit of public health insurance?	*Beneficiați de asigurare publică de sănătate ca:*
		1. High-school student<br>2. College student<br>3. Employee<br>4. Freelancer<br>5. Retired<br>6. Other…	*1. Elev*<br>*2. Student*<br>*3. Angajat*<br>*4. Liber profesionist*<br>*5. Pensionar*<br>*6. Altul…*
**Q4**	Dental treatments covered by public health insurance	What kind of dental treatments did the health insurance authority cover for you?	*De ce fel de tratamente stomatologice ați beneficiat cu decontare prin Casa de Asigurări de Sănătate?*
		1. Periodical check-up and cleanings<br>2. Fillings<br>3. Root canal treatments<br>4. Periodontal treatment<br>5. Dental extractions<br>6. Dental crown/bridge<br>7. Dentures<br>8. Braces<br>9. Oral Maxillofacial surgery<br>10. I don't know<br>11. Other…	*1. Control periodic și igienizare profesională*<br>*2. Obturații*<br>*3. Tratament de canal*<br>*4. Tratament parodontal*<br>*5. Extracții*<br>*6. Coroană/punte*<br>*7. Proteze mobile*<br>*8. Aparat ortodontic*<br>*9. Operații Oro-Maxilo-Faciale*<br>*10. Nu știu*<br>*11. Altul…*

**Q5**	Reasons for usage of public health insurance for dental treatments	On which reasons did you choose dental treatments with included coverage?	*Care au fost motivele pentru care ați ales să faceți tratamentele stomatologice cu decontare prin Casa de Asigurări de Sănătate?*
		1. Financial reasons<br>2. It was the dentist's suggestion<br>3. It was the standard procedure<br>4. It was my right on which I wanted to benefit from<br>5. I have more confidence in these services<br>6. I don't know<br>7. Other…	*1. Motive financiare<br>2. La propuenrea medicului dentist<br>3. Modul de lucru din oficiu<br>4. E un drept de care vreau sa beneficiez<br>5. Am încredere mai mare în aceste servicii<br>6. Nu știu<br>7. Altele…*
**Q6**	Level of coverage of dental treatments using the public health insurance	Were all the dental treatments covered from the health insurance authority?	*În perioada în care ați beneficat de tratamentele stomatologice cu decontare prin Casa de Asigurări de Sănătate, toate tratamentele de care aveati atunci nevoie au fost decontate?*
		1. Yes<br>2. No	*1. Da<br>2. Nu*
**Q6a**	Reasons for coverage for all dental treatments	If YES, please mention why did you choose this option:	*Dacă ați răspuns "DA" atunci va rugăm să menționați motivele*
		1. I covered all the treatments in the insurance budget and it wasn't necessary to wait a period for the treatment start<br>2. I set the treatments on months so that I limit myself to the budget<br>3. I wasn't aware that there was an insurance budget	*1. M-am încadrat cu tratamentele în plafon și nu a fost nevoie să aștept o peioadă pentru realizarea tratamentelor<br>2. Am etapizat tratamentul pe mai multe luni astfel încât să vă încadrați în plafon<br>3. Nu am fost informat/informată cu privire la plafon*
	Reasons for partial coverage	If NO, please mention why did you choose this option:	*Dacă ați răspuns "NU" atunci va rugăm să menționați motivele*

**Q6b**		1. I needed complex procedures and some of them weren't covered<br>2. The treatments I needed were past the insurance budget<br>3. I wasn't informed about the list of covered dental services	*1. Tratamentele de care am avut nevoie au depășit plafonul disponibil<br>2. Am avut nevoie de tratamente complex și unele nu făceau parte din lista celor decontate de CAS<br>3. Nu am fost informat/informată cu privire la plafon sau lista de servicii stomatologice acceptate de CAS pentru decontare*
**Q7**	Patient satisfaction	Were you pleased with the following aspects of the covered dental services?	*Ați fost mulțumit/mulțumită de modul în care s-au desfășurat tratamentele cu decontare prin Casa de Asigurări de Sănătate?*
		1. The types of covered dental services<br>2. The quality of the treatments<br>3. The medical office environment<br>4. The necessary formalities for coverage<br>5. The waiting time for coverage	*1. Tipurile de tratamente decontabile<br>2. Calitatea tratamentelor<br>3. Condițiile din unitatea medicală<br>4. Procedurile de decontare<br>5. Timpul de așteptare necesar pentru decontare*
**Q8**	Self-assessed oral health	How would you describe your current oral health?	*Cum ați descrie starea dumneavoastră de sănătate orală?*
		1. Excellent<br>2. Very good<br>3. Good<br>4. Satisfactory<br>5. Very poor<br>6. I don't know	*1. Excelentă<br>2. Foarte bună<br>3. Bună<br>4. Satisfăcătoare<br>5. Foarte slabă<br>6. Nu știu*
**Q9**	Frequency of tooth brushing	How frequently do you brush your teeth?	*Cât de des vă periați dinții?*
		1. At least twice a day<br>2. Once a day<br>3. Several times a week<br>4. Once a week<br>5. Several times a month<br>6. Never	*1. De 2 sau mai multe ori pe zi<br>2. O dată pe zi<br>3. De câteva ori pe săptămână<br>4. O dată pe săptămână<br>5. De câteva ori pe lună<br>6. Niciodată*
**Q10**	Age	Age	*Vârsta*
**Q11**	Gender	Sex	*Sexul*
		1. Male<br>2. Female<br>3. Other	*1. Masculin<br>2. Feminin<br>3. Altul*
		Last school attended	*Ultima școală absolvită*

**Q12**	Education level	1. High-school<br>2. Professional school<br>3. College<br>4. Post-graduate studies	*1. Liceu*<br>*2. Școală profesională*<br>*3. Facultate*<br>*4. Studii post-universitare*
